# Influence of different jaw positions on dynamic balance using Y‐balance test

**DOI:** 10.1002/brb3.1507

**Published:** 2019-12-20

**Authors:** Hamayun Zafar, Ahmad H. Alghadir, Zaheen Ahmed Iqbal, Amir Iqbal, Shahnawaz Anwer, Ali H. Alnahdi

**Affiliations:** ^1^ Department of Rehabilitation Sciences College of Applied Medical Sciences King Saud University Riyadh Saudi Arabia; ^2^ Department of Odontology Clinical Oral Physiology Faculty of Medicine Umea University Umea Sweden; ^3^ Department of Building and Real-Estate Hong Kong Polytechnic University Kowloon Hong Kong SAR

**Keywords:** dynamic balance, jaw position, static balance, Y‐balance test

## Abstract

**Background:**

Jaw sensory‐motor system has been shown to affect static balance of the body. It would be interesting to know whether it can influence dynamic balance as well. The objective of this study is to examine the influence of different jaw positions on dynamic balance using the Y‐balance test.

**Methods:**

Eighty healthy male participants aged 20–35 years were invited to participate in this study. Dynamic balance was measured by the Y‐balance test in three directions (anterior, posteromedial, and posterolateral) for each leg separately in three jaw positions: resting jaw (control), open‐jaw, and clenched jaw.

**Results:**

There were no significant differences in reach distances between the different jaw positions except in the posterolateral direction. In comparison with resting jaw position, reach distance was significantly higher in open‐jaw position for the right leg and in clenched and open‐jaw positions for the left leg in the posterolateral direction.

**Conclusions:**

Although various studies have shown direct or indirect influence of jaw sensory‐motor system on static postural control, results of this study point to limited relation with dynamic postural control among healthy subjects. However, it supports the potential of the jaw sensory‐motor system to affect motor control during functional tasks in patients with postural instability or similar disorders.

## Graphical abstract

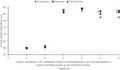

## 1 INTRODUCTION

Isometric contraction of masticatory muscles can affect static balance of the body (Gangloff, Louis, & Perrin, 2000; Sforza et al., 2006). Jaw clenching has been shown to enhance posture stability through facilitation of neural reflexes (Miyahara, 1991; Miyahara, Hagiya, Ohyama, & Nakamura, 1996; Takada, Miyahara, Tanaka, Ohyama, & Nakamura, 2000). Another study has reported improvement in the performance of professional marksmen after using occlusal splints (Gangloff et al., 2000). These effects have been linked to reduction of body sway while jaw clenching (Bracco, Deregibus, & Piscetta, 2004; Bracco, Deregibus, Piscetta, & Ferrario, 1998; Sakaguchi et al., 2007). It has been further supported by various studies on animal models which show neuronal connection between trigeminal nerve, brainstem nuclei, and spinal cord (Devoize et al., 2010; Ruggiero, Ross, & Reis, 1981). Modification of jaw position also affects neck muscle endurance (Zafar, Alghadir, & Iqbal, 2019).

Added advantage of dynamic balance assessment is the ability to assess components of strength, proprioception, and range of motion to the ability of maintaining steady and upright position (Gribble & Hertel, 2003; Paterno, Schmitt, Ford, Rauh, & Hewett, 2013). Such purposeful and task‐oriented body movements involve combined action of different joints, muscles, and nerves (Zafar, Nordh, & Eriksson, 2002). The Y‐balance test (YBT) is the instrumented version of the modified Star Excursion Balance Test (SEBT) that has been regarded as reliable and valid measure to evaluate dynamic balance (Alnahdi, Alderaa, Aldali, & Alsobayel, 2015; Gribble, Hertel, & Plisky, 2012; Hyong & Kim, 2014; Plisky et al., 2009; Plisky et al., 2009; Shaffer et al., 2013). It measures reach distance in anterior, posteromedial, and posterolateral directions with one leg while standing on other leg (Plisky et al., 2009). It is also an aid to identify lower‐extremity flexibility deficits, asymmetries, and impairments (Endo & Sakamoto, 2014b; Gribble et al., 2012; Hyong & Kim, 2014; Lee, Kim, Ha, & Oh, 2014; Overmoyer & Reiser, 2015), as well as a screening tool to predict injury risk at the cause of poor neuromuscular control (Butler, Lehr, Fink, Kiesel, & Plisky, 2013; Endo & Sakamoto, 2014a; Plisky, Rauh, Kaminski, & Underwood, 2006; Smith, Chimera, & Warren, 2015).

Having established direct or indirect links between jaw sensory‐motor system and static balance (Alghadir, Zafar, Whitney, & Iqbal, 2014; Alghadir, Zafar, & Iqbal, 2015), it would be interesting to know whether it can influence dynamic balance as well. This study was done to see the influence of different positions of jaw on dynamic balance. We studied the effect of three jaw positions, that is, resting jaw, open‐jaw, and clenched jaw on dynamic balance using YBT. We hypothesized that variation in jaw sensory‐motor system can affect reach distance significantly among healthy adults.

## 2 METHODS

### 2.1 Participants

Eighty healthy male participants aged 20–35 years were invited for this study. Any case of musculoskeletal injury in the last 1 year, back pain in last 6 months or history of surgery or temporomandibular joint disorders, or any other neurological problem was excluded. All participants were briefed about the need of the study and asked to sign a consent before participation. An ethical approval according to Declaration of Helsinki was obtained from research committee of our institution.

Participants' data including age, weight, height, and leg length were recorded. Leg length for the dominant side was measured from the anterior superior iliac spine to the most distal part of the medial malleolus in supine position (Plisky et al., 2006).

### 2.2 Dynamic balance

Dynamic balance was measured using YBT (Move2Perform, Evansville, IN). Test was conducted as described in previously published studies (Alhusaini et al., 2017; Alnahdi et al., 2015; Plisky et al., 2009; Smith et al., 2015). Barefooted participants were advised to perform practice trials before actual data collection. YBT was conducted in three positions: resting jaw (natural jaw position with no instructions; control), open‐jaw (jaws slightly apart with no contact between tooth), and clenched jaw (jaws tightly closed against each other). The order of the test was random. Three trials were recorded for both legs in each direction. The participants were asked to stand on one leg and reach the indicator as far as they could by using other leg and then return to the starting position without losing their balance. Reach distance was recorded to the nearest 0.5 cm. The trial was repeated if participants failed to return to the starting position without losing balance or they kicked the indicator. The mean of three trials was used for data analysis. Normalized reach distance was calculated by dividing this value by limb length and multiplying by 100 (Gribble & Hertel, 2003).

### 2.3 Statistical analysis

Data were analyzed using GraphPad Instat 3.0 software. Mean, standard deviation (*SD*), and 95% confidence interval were presented, and hypothesis of no difference in reach distance between 3 positions was tested by parametric repeated measures ANOVA using Bonferroni multiple comparisons test, and it was rejected if *p* value was <.05.

### 2.4 Ethics approval 

All subjects were informed about the aims and procedures of the study, and written informed consent was obtained for participation in the study. This study was approved by the Rehabilitation research review board for ethics according to Declaration of Helsinki (Ref no. KSU/RRC/031/01).

## 3 RESULTS

After passing the inclusion and exclusion criteria, 59 healthy male subjects participated in this study. Demographic data have been presented in Table 1. Actual and normalized reach distance values for the YBT are shown in Tables 2 and 3, respectively.

**Table 1:** Demographic characteristics of the participants

Variable	Mean (*SD*)
Age (years)	25.77 (5.95)
Height (cm)	171.81 (6.59)
Weight (kg)	75.62 (18.36)
Leg length (cm)	92.20 (4.44)

Abbreviation: *SD*, standard deviation.

**Table 2:** Actual reach distance values for the Y‐balance test (cm)

Variable	Resting position		Open‐jaw position		Clenched jaw position	
	Mean (*SD*)	95% CI	Mean (*SD*)	95% CI	Mean (*SD*)	95% CI
Right anterior	59.45 (6.58)	57.74–61.17	59.94 (7.2)	58.05–61.82	60.07 (6.83)	58.29–61.86
Left anterior	61.27 (6.91)	59.47–63.08	61.36 (7.4)	59.42–63.30	60.44 (6.90)	58.63–62.26
Right posteromedial	86.42 (9.51)	83.94–88.91	87.93 (10.71)	85.13–90.72	86.30 (10.75)	83.50–89.10
Left posteromedial	87.36 (9.46)	84.89–89.82	89.02 (10.57)	86.27–91.78	88.94 (9.81)	86.39–91.50
Right posterolateral	82.53* (11.67)	79.49–85.58	86.87* (11.76)	83.80–89.93	85.23 (12.5)	81.95–88.51
Left posterolateral	82.53* (11.10)	79.63–85.42	87.34* (11.72)	84.28–90.40)	86.53* (10.74)	83.73–89.33

* Significant difference *p* < .05.

**Table 3:** Normalized reach distance values for the Y‐balance test (expressed as percentage of leg length)

Variable	Resting position		Open‐jaw position		Clenched jaw position	
	Mean (*SD*)	95% CI	Mean (*SD*)	95% CI	Mean (*SD*)	95% CI
Right anterior	64.49 (6.42)	62.81–66.16	65.05 (7.54)	63.08–67.02	65.17 (6.90)	63.37–66.98
Left anterior	66.53 (7.57)	64.56–68.50	66.59 (7.70)	64.58–68.60	65.59 (7.22)	63.71–67.48
Right posteromedial	93.82 (10.21)	91.16–96.49	95.30 (10.17)	92.65–97.95	93.57 (10.54)	90.82–96.32
Left posteromedial	94.87 (10.49)	92.14–97.61	96.55 (10.54)	93.80–99.30	96.51 (10.00)	93.90–99.12
Right posterolateral	89.50* (11.77)	86.43–92.57	94.17* (11.65)	91.14–97.21	92.45 (13.086)	89.04–95.86
Left posterolateral	89.56* (11.69)	86.52–92.61	94.75* (12.12)	91.59–97.91	93.90* (11.18)	90.98–96.81

* Significant difference *p* < .05.

### 3.1 Resting jaw position

Actual reach distance values in the right anterior, posteromedial and posterolateral directions were 59.45, 86.42, and 82.53 cm, respectively, while in the left anterior, posteromedial, and posterolateral directions were 61.27, 87.36, and 82.53 cm, respectively.

Normalized reach distance values in the right anterior, posteromedial, and posterolateral directions were 64.49, 93.82, and 89.50%, respectively, while in the left anterior, posteromedial and posterolateral directions were 66.53, 94.87, and 89.56%, respectively.

### 3.2 Open‐jaw position

Actual reach distance values in the right anterior, posteromedial, and posterolateral directions were 59.94, 87.93, and 86.87 cm, respectively, while in the left anterior, posteromedial and posterolateral directions were 61.36, 89.02, and 87.34 cm, respectively.

Normalized reach distance values in the right anterior, posteromedial and posterolateral directions were 65.05, 95.30, and 94.17%, respectively, while in the left anterior, posteromedial and posterolateral directions were 66.59, 96.55, and 94.75%, respectively.

### 3.3 Clenched jaw position

Actual reach distance values in the right anterior, posteromedial and posterolateral directions were 60.07, 86.30, and 85.23 cm, respectively, while in the left anterior, posteromedial and posterolateral directions were 60.44, 88.94, and 86.53 cm, respectively.

Normalized reach distance values in the right anterior, posteromedial and posterolateral directions were 65.17, 93.57, and 92.45%, respectively, while in the left anterior, posteromedial and posterolateral directions were 65.59, 96.51, and 93.90%, respectively.

### 3.4 Comparison between three jaw positions

Although reach distance (actual as well as normalized) varied between the three jaw positions, there were no significant differences in all directions except the posterolateral direction.

**Figure 1:**
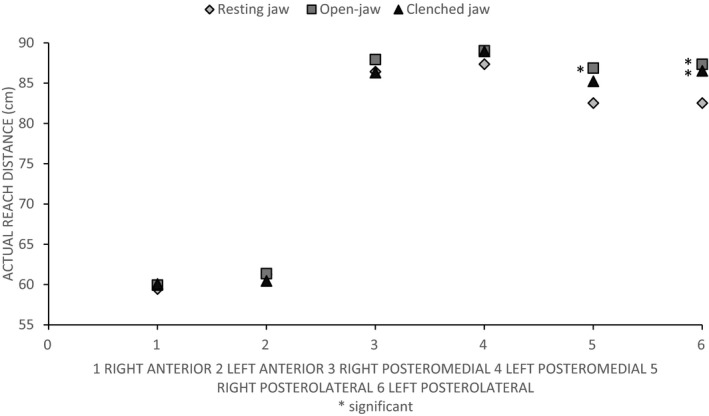
Actual reach distance values for the Y‐balance test (Mean, cm). Note significantly higher values in open‐jaw position for the right leg and in clenched and open‐jaw positions for the left leg in the posterolateral direction significant in comparison with resting jaw

**Figure 2:**
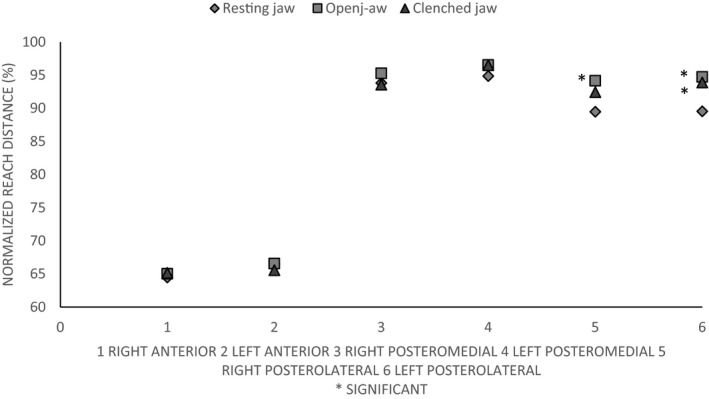
Normalized reach distance values for the Y‐balance test (%). Note significantly higher values in open‐jaw position for the right leg and in clenched and open‐jaw positions for the left leg in the posterolateral direction significant in comparison with resting jaw

For right leg, actual and normalized reach distance values were significantly higher in open‐jaw position in the posterolateral direction as compared to resting jaw position(*p* < .05). For left leg, actual and normalized reach distance values were significantly higher in clenched and open‐jaw positions resting jaw position (*p* < .05) as compared to resting jaw position (Figures 1 and 2).

## 4 DISCUSSION

The purpose of this study was to see the effect of three different jaw positions, that is, resting jaw, open‐jaw, and clenched jaw on dynamic balance using YBT. Although reach distance varied between the three positions, in comparison with resting jaw, it was significantly higher in open‐jaw position for the right leg and in clenched and open‐jaw positions for the left leg in the posterolateral direction only.

Postural control is static (when attempting to maintain a position with minimum movement) or dynamic (involving completion of a task during movement without compromising base of support) (Gribble & Hertel, 2003; Winter, Patla, & Frank, 1990). It is a complex system that depends on information from the proprioceptive, vestibular, ocular systems, and neck reflexes (Horak, 2006). The role of visual input and standing surface on balance control is well documented (Alghadir et al., 2015; Mohapatra, Kukkar, & Aruin, 2014; Redfern, Yardley, & Bronstein, 2001). Influence of jaw sensory‐motor system on vestibular, neck, and ocular systems has been shown (Alghadir, Zafar, Iqbal, & Al‐Eisa, 2018; Davies, 1979; Ehrlich, Garlick, & Ninio, 1999; Hellmann, Giannakopoulos, Blaser, Eberhard, & Schindler, 2011; Park et al., 2014), and thus, it has the capacity to affect posture control. Variation in activation pattern of the jaw sensory‐motor system while maximum biting, sub‐maximum biting, clenching or chewing has been shown to modulate strategies of central postural motor control mechanisms differently (Alghadir et al., 2014; Hellmann et al., 2011; Kushiro & Goto, 2011). These include improvement in sports performance, distal muscle strength, and postural balance (Cherry, Brown, Coburn, & Noffal, 2010; Hosoda et al., 2007). Instant reduction of body sway after using dental splints in patients with whiplash‐associated disorders in comparison with healthy subjects further supports the conspicuous role of jaw sensory‐motor system (Eriksson, Zafar, & Backén, 2018). Therefore, it can be postulated that the influence of jaw positions on dynamic balance is expected to be more in patients with postural instability or similar disorders rather than healthy subjects.

Although posteromedial component of the SEBT has been shown to highly represent the performance in all its components (Hertel, Braham, Hale, & Olmsted‐Kramer, 2006), our results show significant differences in reach distance values in posterolateral direction with open‐jaw and clenched jaw positions. Posteromedial and posterolateral reach distance have been positively associated with hip abductor strength (Hubbard, Kramer, Denegar, & Hertel, 2007; Lee et al., 2014). Larger hip range of motion is needed while reaching in posterior direction (Robinson & Gribble, 2008). Challenging balancing tasks lead to modification of fusimotor drive and muscle tone (Aniss, Diener, Hore, Gandevia, & Burke, 1990). Such mechanisms could bring the most symmetric neuromuscular equilibrium during open‐jaw and clenched jaw positions (Gangloff et al., 2000).

Y‐balance test is the instrumented version of the modified SEBT, and its performance has been shown to vary among different cultures (Butler, Queen, Beckman, Kiesel, & Plisky, 2013; Plisky et al., 2006; Smith et al., 2015). Reach distance values from SEBT have been also shown to be associated with leg length and its normalization or matching paired participants for leg length have been recommended (Gribble & Hertel, 2003). However, same results were found in the analyses of the actual and normalized reach distance values for the leg length in this study.

## 5 CONCLUSIONS

Although various studies have shown direct or indirect influence of jaw sensory‐motor system on static postural control, results of this study point to limited relation with dynamic postural control among healthy subjects. However, it supports the potential of jaw sensory‐motor system to influence motor control during functional tasks in patients with postural instability or similar disorders and further study is recommended.

## CONFLICT OF INTEREST

None declared.

## AUTHOR CONTRIBUTIONS

HZ and AA proposed research idea and design. AA, AHA, and ZI reviewed the manuscript. ZI, AI, and SA executed data collection and analysis. HZ, SA, AI, and ZI prepared and submitted the manuscript.

## Data Availability

The datasets used in this study are available from the corresponding author on request.
